# Abstracts and Gleanings

**Published:** 1888-06

**Authors:** 


### ABSTRACTS AND GLEANINGS.

**A very Valuable Lesson for those who use Anæsthetics.**— In a paper read before Balt. Academy of Medicine by Julian J. Chisholm, M. D., the following case is given:

R. A., a robust, healthy child, three years of age, was recently brought to me with a cancerous left eye. The attention of the parents was first called to the yellow appearance of the pupil eighteen months before. The gliomatous mass filled the vitreous cavity, distending the pupil and obliterating the anterior chamber. The eye was injected and painful. The prompt removal of the eyeball was urged as the only means of protecting the child from a painful death. The operation was accepted by the parents, and the enucleation, under chloroform, accomplished under much difficulty, as the sequel will show.

The child was suffering from a bronchial trouble, but that was not deemed an obstacle to the administration of an anæsthetic. The patient was placed on the operating-table, his clothing loosened about the neck and chest, and chloroform was inhaled from a towel, folded in conical form, with open top. Deep sleep was soon induced.

When the anæsthesia was complete, the operation for the removal of the diseased eye was commenced. The conjunctiva was divided around the cornea, and the tendon of the external rectus muscle was being sought for, when respiration suddenly ceased. The face assumed a death-like pallor, the pulse disappearing at the same time from the wrist. Immediately the child was suspended by the feet, with body and feet hanging down at an inclination of seventy degrees, while an assistant volunteered chest-compression for artificial respiration. After a few minutes, signs of a feeble respiratory movement were noticed, a slight throbbing of the neck-vessles was detected, and in time the child evinced its return to conciousness by crying.

He was laid on the table, but would not permit the eye to be touched without a twist of the head, evincing great irritability or sensitiveness of the conjunctiva. As the operation had to be completed, I ardered chloroform to be administered. Chloroform narcosis was very soon re established, but before I had time to resume the operation the child again stopped breathing and the pulse disappeared. The body, apparently of a dead child, was once more hung up by the feet, so as to allow blood to gravitate toward the anæemic head and brain, but with no further attempts at artificial respiration. Myself and four assistants watched anxisusly the pale face, to catch the first evidence of returning vitality. After some minutes I noticed that the large vessles at the root of the neck showed some fulness, then a slight thrill, and after this the first attempt at a thoracic movement appeared. In ten minutes breathing was sufficiently strong to allow the child to cry again, much to the relief of all of us.

Still the operation which was so imperitively called for, for the future safety of the child—even the saving of its life from the ravages of cancer—was uncompleted. While the father and mother, both present in the operating-room, were pleading for their child, they were made aware, by the restlessness of the patient when the eye was touched, that nothing could be done without the child going again to sleep, so I once more ordered the inhalation of chloroform. For the third time chloroform narcosis was prrmptly established, and was followed very soon afterwared by suspended respiration and the disappearance of the pulse. Death now seemed to be complete. Immediately the child was hung up by the feet. The absolute quiet of the operating-room was broken only by the lamentations of the parents. All eyes watched the face of the child. Five minutes seemed an hour, and the ashy lips showed, so far, no response. Soon after this a faint effort at respiration was observed, which became stronger with each return of the thoratic movements, and the pulse was again felt feebly at the wrist. When respiration seemed established, complete insensibility continueing, I had the child laid upon the operating-table. As soon as the body assumed the horizontal position, the pulse, not yet strong, disappeared from the wrist, and the respiration ceased, necesitating at once a renewal of the suspension. This curious phenomenon of breathing when suspended, and becoming inanimate when the prone position was too early assumed, was repeated two or three times, respectively. For safety—for I was afraid to lay the child down—I was forced to enucleate the eye while the child was suspended with head downward, an awkward position for operating. It was some time, fully a quarter of an hour, after the operation was completed and the eye bandaged, before I could trust the child in the recumbent posture.

One of my assistants was very anxious to have whisky injected, and had filled his hypodermic syringe for that purpose; but I declined its use, trusting to inversion alone for resusitation. The final successful issue of this case confirmed my faith in this invaluable method, which I had used successfully on former occasions, and hence confided in it for the protection of the patient through the trying ordeal. In all, the child must have been suspended in the inverted position for fully three-quarters of an hour. After the last suspension no further trouble ensued. The next day the child was so thoroughly himself that he left the hospital with his parents. He was brought back to the dispensary, for inspection, two days afterward, a picture of health.

This case cannot be tooo carefully studied by surgeons who must continue to use general anæsthetics. It is one of a series occurring to me now and then—I am glad to say at long intervals—as the consequence of chloroform inhalation.

I am a strong advocate of chloroform, believing it to be the most available remedy of its class. I recognize it as a powerful agent for evil, but at the same time I believe it to be the best of the general anæsthetics. In army life and in civil practice I have had a personal experience of at least ten thousand administrations, and without a death. For thirty years I have had charge of a surgical hospital service, and my daily use of chloroform has been the subject of public professional observation. Sulphuric ether I have seldom used—not one hundred times in my life, and in most of these instances only to exhibit on patients the effects of the various anæsthetics to medical classes at the University of Maryland Hospital Clinic. In the last ten years I have not used it once. For painful operations of very short d ration I use the bromide of ethyl, and for all others I use chloroform exclusively.—*Med. Herald.*

**Cholecystotomy with Recovery.**—On February 14, M. Polaillon reported to the Academy of Medicine of Paris a case of cholecystotomy by M. TERRILLON, that was followed by recovery.

The patient was a woman, æt 24, who had had no previous liver trouble, until she noticed a tumor in the right side. On examination M. Terrillon made a tumor, below the border of the ribs, that extended to below the umbilicus. The skin was normal, the umbilical cicatrix regular, and there was no enlarged subcutaneous veins. Palpitation showed the tumor to be as large as the head of a fœtus, tense, elongated vertically, smooth, and very movable transversely. It did not appear to follow the respiratory movements. Its extent was easily made out by percussion. Its dullness was coextensive with that of the liver. In other respects abdominal percussion was normal. There was no icterus, no pain, and no serious inconvenience of any kind. There was some dyspepsia and emaciation. The urine was normal. It was evident that the tumor was connected with or adherent to the liver. No exploratory puncture was made, but Terrillon determined to make an exploratory laparotomy.

Operation on November 23, 1886. A median vertical incision was made, about 4 cm. long, and afterwards enlarged to 8 cm. When the abdomen was opened the lower border of the liver was easily recognized, since it was lower than normal. At the level of the lower border, and attached to the inferior face, was an elongated tumor, which descended very low, had bluish walls, was very fluctuant, and of a cystic appearance. The finger introduced into the wound showed that the tumor was a distended gall-bladder. Puncture with a capillary trocar gave issue to a liquid as clear as water, but a little thicker, and the last drops of which were of a milky whiteness. It was then found that in the gall-bladder was a gall-stone as large as a cherry. The bladder was now drawn partly out of the abdominal wound, and then opened. Its walls were very vascular. Hæmorrhage was guarded against by forceps, and the cavity was explored.

The first calculus found was easily extracted, though it was adherent to the bladder by a thin filament. The finger carried into the bladder then found, at the far side, the summit, of the cavity, a second calculus imbedded in the mucous membrane. It was so deeply imbedded that, at the distance it was from the opening, it was impossible to detach it.

The bladder was then drawn further out, so as to lessen the depth of the cavity, when the stone was removed in fragments by means of forceps.

Having resected a portion of the base of the bladder, the operator sutured it to the abdominal wall, making a large biliary fistula in which two large drains were placed, and a dressing then applied.

In about a month the fistula was so far closed as to admit only a very small filiform bougie. Bile flowed abundantly from this orifice, generally between 9 p. m. and 2 a. m.—about four hours after the last meal. Two cauterizations with the thermocautery caused the fistula to close completely, two months after the operation. Meanwhile the patient had gained flesh, and was completely cured.

In commenting upon the case M. Polaillon says that, generally, the most favorable incision for cholecystotomy is one following the external border of the rectus muscle. B t in order to have more room one must som[illegible]times add to this a more or l[illegible]ss transverse incision a little bel[illegible]w the bor[illegible]ler of the costal cartilages. M. Terrillon's incision, however, was for an exploratory laparotomy.

Opening of the gall-bladder may give rise to hæmorrhage, which must be controlled by hæmostatic forceps, or to extravasation of of bile, which must be prevented at all hazards by drawing the bladder out and protecting the peritoneum by aseptic sponges. Suture of the abdominal wall completes the operation in cases in which there is no calculus. When a biliary fistula is established the more pressing danger is passed, and intraperitoneal rupture of the gal.-bladder is prevented.—*Bulletin d l' Academie de Medicine*, No. 7, 1888.

**The Tteatment of Sleeplessness**.—Eccles (*Practitioner*, March, 1888) describes the various methods which he has employed during the past two years to produce sleep. Massage is a very valuable means of treatment to restore the habit of sleeping; but the object of this paper is to describe, outside of narcotics, the means used to produce an immediate effect. These means have been long and familiarly known, but their respective aplicability to nifferent disorders of sleep does not seem to be so well understood; and it is true that those which are adapted to one class of cases, may be useless or even harmful to another class. Patients who "constantly dream" and have but restless unrefreshing sleep, are often treated in various ways in the effort to overcome the partial functional activity of the brain. The best treatment is probably a hot bath taken immediately before the individual wishes to settle quietly for the night. To make it successful it is necessary that even the petty details be attended to. The room should be of a temparature of not less than 65° F., and the patient should be stripped and the head then douched with water at 100° F.; this chilling of the surface and heating of the head filling the brain with blood. The whole body of the patient, excepting the head, is then immersed in a bath of 105° to 110° F. for eight to fifteen minutes; then wrapped in blankets, taken to the bedroom, dressed in night clothes and put to bed with a hot bottle to the feet and the head well raised. In this way the *whole of the brain* undergoes a reduction of blood supply. and dreaming is psevented. The contra-indications to the hot bath are extreme emaciation or anæemia, atheroma, and aortic valvular disease. In these conditions a stimulant rather than a depressant to the nervous system is required, and Brunton has, therefore, recommended strychnia as a hypnotic.

Where there is organic heart disease or functional weakness of the heart and circulation, general corporeal massage at night will frequently produce sleep, particularly if precautions be taken that the surface of the body be not chilled. In certain other cases of insomnia depending on an overwrought and enfeebled nervous system there is hyperæsthesia, and vigorous kneading cannot be employed with benefit. In these instances gentle stroking of the back and limbs must be substituted; or gentle kneading of the the abdomen, followed by a hot abdominal compress. This compress consists of two bandages four yards long, and one and a half yards wide; heated dry in a hot oven, and brought to the bedside in a covered jar. The end of one of them is dipped in water for as much of it as is necessary to cover the abdomen, applied to the surface, and the rest of the bandages wound around this. In still other cases of insomnia from prolonged overwork, mental distress, the morphia or chloral habit, etc., a condition appears in which massage of any sort applied in the evening must be omitted, since it only tends to increase the nervous excitement. Prompt action is necessary, yet drugs are useles. Under these circumstances there is no agent so useful as the wet pack carefully administered. The patient should remain in it twenty minutes to half an hour, and then, on opening the pack, should be quickly clothed in previously warmed night-clothes, and well covered in bed. Where the action of the pack is very rapid and transitory, its coverings should be sooner and more gradually removed, and the patient not allowed to become too warm in it.

By some one of these methods, followed by recumbent rest in bed, and absolute quiet, sleep may almost invariably be produced. The action is, however, only temporary, and for permanent restoration some other and radical treatment is required. Continued recumbent position in bed in a quiet room, away from social, business, or domestic cares, a carefully modified diet, and massage properly applied will, in the majority of cases, at last be followed by the happiest results.

**Antipyrine and Antifebrin.**—DR. E. EVANS in a communication to the *Canada Med. and Surg. Journal*, Feb., 1888, remarks that the following notes may prove of interest, as illustrating not only the anodyne powers of antipyrin and antifebrin in eertain painful affections, but also their *curative* powers. It is said that neuralgic affections attacking persons in the period of declining bodily vigor are often severe, lingering, and rebelious to treatment. The following cases occurring at this period yielded readily to one or the other of the above drugs:—

CASE I.—J. E., farmer, aged 65; had worked hard in harvest field and felt exhausted; complained of pain in right hypochondrium and scanty micturition. Was seen by a doctor, who prescribed rest, a purge, and a mild diuretic mixture. Two days later Dr. Evans found him still suffering severely with pain, but in addition, almost total suppression of urine, and frequent desire to micturate. He was drowsy, had a headeache, and vomiting.

Pulse, 115; temperature, 102 5°. One and a half ounces of urine were drawn off, which was dark; great excess of urates; specific gravity 1.035; only a trace of albumin; no blood or tube casts. Gave him hot bath and pulv. Doveri gr. x and pilocarp. gr. 1/6 (bowels were open), and poulticed the joins. The next day the headache was gone, vomiting ceased, and no frequency of miciurition; pulse and temparature normal; urine passes in small quantity and contains no albumin. In three days he was well again, except being very weak. The pain in his side persisted, however, and was very severe. It was confined to the ninth and tenth intercosspaces (tender points present). Blisters, hypodermics of morphia and atropia, and the use of arsenic failed to give more than temporary relief, and he had to take opium constantly for a month to obtain sleep. Antipyrin grs. v twice daily, was then tried, all other treatment being stopped. He had immediate relief, and in ten days was entirely well.

CASE II.—T. M. aged 54, soldier, suffering from intercostal neuralgia (ninth interspace, right side). Pain very severe, intermittent, tender points, ten days' duration. A history of a similar attack fifteen years ago, after severe marching. No rheumatism, no syphylis. A hypodermic (gr. viii) of antipyrin gave immediate relief. Antifebrin gr. vi twice a day was prescribed, and liquor arsenicalis m iii t i. d. He suffered no pain since and is now well.

CASE III.—Mrs. H., aged 48, dorso-lumbar neuralgia, a large fat woman with rheumatic history. She had ill-defined paines in the back and hips for some time, when, after "catching cold," she was suddenly seized with severe pain in the lumbar region, which confined her to her bed. Pain much increased by stirring, and radiates over butt cs and into hypogastric region. Tender points in lumbar region, over centre of crista ilii and in hypogastrium. A hypodermatic injection of gr. x of antipyrin stopped the pain, and in ten minutes she could move freely in bed without pain. He left four powders of gr. v antipyrin' and she has not suffered from pain since then.

He has also used these drugs for the pains of locomotor ataxia, (one dose of gr. x antipyrin stopped the gastric pain of ataxia for three months), cervicobrachial neuralgia, painful affections of the fifth nerve, four cases migraine, dysmenorrhœa, gastralgia, intestinal colic, etc. In fact in almost all cases where formerly morphia was used to relieve pain.

So far he has found antipyrin fail in one case of migrane and in a case of hysterical pain. It seems to posess the advantage over morphia of not deranging the digestion or producing other untoward effects. Smaller doses than those generally prescribed, will, he thinks, be found equally effective, and besides there is little danger of excessive sweating, vomiting or collapse. Antifebrin seems equally effective as antipyrin to relieve pain. It is about four times as cheep, and the dose is smaller; but it is not so soluble, and is not therefore so suitable for hypodermic use.

**Antipyrine for Headaches.**—Since the introduction of our therapeutic list of antipyrine, and its application as a remedy for cephalalgia, hopes have been held out that an almost certain, specific remedy has been discovered for that very general and burdensome complaint. As regards the dose and mode of administration. we cannot do better than give the method of Germain See. who has awakened such great interest in the subject in Paris. He says: Starting from these data, and knowing the depressant effects of antopyrine on the excitability of the cerebro-spinal system, I have deemed it rational to subject my patients to the use of this mendicament, of whose calmative and analgetic properties I have now had such striking evidence. Antipyrine is given from the outset of the attack; fifteen grains on waking in the morning, and fifteen grains an hour after. In all, without exception, after the second dose, or a little later, the paroxysm, which was wont to last all day, and even through the night, has been arrested, and the patients have been able to resume their studies, or their usual occupations. In every instance the remedy has been given in a half a tumblerful of cold water just before or during the morning meal; the pain has usually disappeared in twenty or thirty minutes, the second dose acting only the part of a preventive.—*Review.*

**How to Tampon**.—Dr. Valin (*Medical Age*, April 25, 1884.) writes: The classical tampon requires a great quantity of material; 30 to 40 little balls of lint, of the size of a walnut, impregnated with some fatty substance, *e.g.*, carbolized vaseline are necessary. The first 15 or 20 are tied by a strong thread, which facilitates their withdrawal. The quantitity of material required to make a good tampon is apt to astonish the young physician. But the physician readily understands that it is a question not only of filling the vagina, but also of the pelvic cavity. The vagina, too, is capable of a great dilatation, and consequently, if the tampon is insufficient *i. e.*, if it does not distend the vagina to its maximum, the blood coming from the uterus will make its passage between the vaginal walls and the periphery of the tampon. Vaseline, or some fat substance, is necessary, in order to secure the closest co-operation of the many little balls of lint, thus making a solid, close and compact body. The safety it procures is absolute. It is not necessary to soak the balls of lint in an astringent liquid, this procedure being injurious. The tampon is not designed as an astringent agent, but a purely mechanical one, the great power of which resides in the fact that it opposes an insuperable dike to the flooding blood. To saturate it with an astringent is apt to corrode or cauterize the vaginal mucous membrane, and thus inflict useless suffering. Moreover, it is easy to understand that the astringent livuid is useless when applied with a tampon, because it (the tampon) is not introduced within the uterus—the origin of the flooding.

To properly apply the tampon, the patient must be placed in the obstetrical position. We begin by emptying the bladder, rectum and vagina, if the case is not too urgent. The speculum is not needed at all. I introduce the balls of lint, well impregnated with carbolized vaseline, the one after the other, packing them well with the index finger at the bottom of the vagina in order to fill every interstice or space. After having made as [illegible] and solid a rampart

I close the anteria vaginal space with wadding or lint. The vagina must be so filled up that the perineum is rendered convex by the pressure coming from inside and the vulva must be also open. Lastly, I apply on the genital parts a good quantity of lint, making a cushion, and keep the whole in place with a T-bandage. This is the classical and real tampon, and the only good one. Every other tampon is not good and consequently dangerous on account of the false security it gives.

The tamponing after delivery is very dangerous, because the blood, not finding an exit, is accumulated in the nterus, which may be distended to enormous proportions It is much better in such a circumstance to give ergot hypodermically, to make frictions on the womb in order to promote or excite uterine contraction. Ergot is under these conditions most indicated, and is a capital remedy. But if the hemorrhage continues despite the introduction of the hand in the uterus to excite contractions, the frictions and the administration of ergot, we must make intra-uterine injections of hot water with persulphate of iron. Here the hot water excites the uterine contraction, coagulates the blood, and is helped in this way by the styptic action of the persulphate of iron, which acts directly now on the flooding blood-vessels.

**Good Daily Diet.**—*Morning. 1st. Breakfast:* Coffee, tea or cocoa (with or without well-boiled milk) eggs, bread (that has been well heated and dried in an oven or stove for ½ hour [in other words, bread that has been cooked a second time] *without* butter.

*2d. Breakfrst:* Boullion with egg, bread as above, warm meat, wine.

*Noon meal:* Hot soup, boiled or stewed meat, roast meat, vegetables, fresh cooked compote, red wine or good beer.

*Tea:* Coffee or tea.

*Supper:* Tea; or soup made from the meat left over from the noon meal, with the morning's bread, or warm meat. Wine or beer as above.

5. Every irregularity of the body should be most strictly guarded against in time of cholera. Apparently slight diarrhœa should not be neglected, but a physician should be consulted immediately.

Sachse suggests that in case of threatened invasion of cholera these rules should be printed in the newspapers, journals, etc, in addition to being printed on slips for public distribution. [The more important parts of these rules should be printed in bold black-faced type. The large foreign element in America would render it necessary that these rules be printed in other languages than English. For the German, the rules may be copied *verbatim* from the original].—*Deutsche medicinische Wochenscrift*, Decem. 22, 87.

**Tribrimphenol.**—A new substitution product, in which bromine and carbolic acid are combined, has recently been added to the very numerous agents already before the medical profession (*Deut. Med. Wochen.*, quoted in *Therepute. Monatshfte* for March 1888.)

Dr. F. GRIMM finds tribromphenol an efficient antiseptic in skin diseases. It is slighty soluble in winter. It is not irritating to the skin. Topically, it is an efficient application to wounds, to atonic granulations, and strumous affections of the mucous membranes. It remains to be shown how effective it is as a poison to bacteria.

Although fitted especially for topical use, it promises well as an internal remedy in affections of the stomach and intestines.

**Sulphate of Codeine.**—I have been prescribing this medicine and carefully observing the results for the last four years, and for me it has proved to be a valuable remedy. I have often wished for a medicine that had good anodyne and sedative proper ties, which would be less objectionable and act more kindly with some of my patients than sulph of morphia. I know by experience that I have found it in sulph. of codeine,—without the usual disagreeable after-effects produced by morphine: such as nausea, dry tongue, and disagreeable feeling about the head.

I first began using it as a sedative in cough prescriptions in phthisis, bronchitis, and for the cough that usually follows attacks of asthma. I often use a prescription like this:

℞. Syr. ipecac,
Syr. scilla, .......................... aa. ℥ ij.
Sulph. codeine, .......................... grs. iii. M.
S. Teaspoonful ior anadult every four hours.

We can always make up the precscription with syrups to suit the case. When I have a patient with a persistent hacking cough with considerable irritation, that don't yield to cough syrups—I give the following:

℞. Pulv. ipecac, .......................... grs. vi.
Pulv. gum acacia, .......................... grs. xl.
Sulph. of codeine, .......................... grs. ij. M.

Divide into twenty powders and take a powder on the tongue dry (swallowing without water) every four hours.

I know by experimenting that the powders act more promptly and effectually than any cough syrup does or can. They give the same constitutional benefit that a syrup can give. Besides they have a more direct local influence, and in this way I manage to control the most obstinate coughs very satisfactorily.

I use it quite extensively for excesive pain in various diseases when an anodyne is indicated, and I almost always get the effect as quickly and permanently as I do from morphine. I have some patients that will not take morphene if they know it; they say that the nausia and depressed feeling that follow ten hours after taking a large dose are about equal to the pain. To them I give sulph. of codeine, which relieves their suffering conditions, satisfies their mind, and makes it pleasant for me.

I have been giving about the same sized dose that I do for morphine, but have given a little larger dose without producing any bad effect.

Perhaps the other members of the association have tested this medicine more thoroughly than I have, and if they have I would be glad to hear from them.—*Eclectic Medical Journal.*

**Antifebrin as a Nervine.**—Dr. Paul Demieville, of Lausanne, (British Medical Journal), has used the drug in eleven cases of sciatica. eight of lumbago, seven of intercostal and mammary neuralgia, eight of trigeminal neuralgia, eleven of headache (megrim, dyspeptic, and chloratic cephalalgia, etc.,) three of neuralgia of the forearm and hand, seven of painful menstruation, two of senile gangrene, three of tabes, six of epilepsy, cancer, nettle-rash, hepatic colis, etc. To adults he gave as a rule half-gramme doses (seven and a half grains), from one to four times daily; if that failed, three-fourths of a gramme, and one gramme doses (fifteen grains) were given once, twice, or thrice a day. On the whole, Dr. Demieville is pleased with antifebrin as an anodyne The pain usually disappears within a period varying from a quarter of an hour to two hours. Its sedative action is very often accompanied by decided hypnotic effect, which is extremely welcome in cases in which pain is associated with obstinate sleeplessness. In some cases the anodyne effects are only temporary. But in many cases a complete cure is effected within a couple of days or so. As an anodyne, this new drug has been especially valuable for the relief of the agonizing pain of senile gangrene and cancer. Antifebrin also seems to diminish the frequency of epileptic fits. He recommends it also in hysterical fits and in infantile convulsions. Dr. Demieville states that he has never known it to produce rigors or noises in the ears; but he has sometimes observed diaphoresis and slight giddiness, and once (in a drunken epileptic aged fifty-one) delirium and hallucinations, resembling those of salicylism. As a rule, the drug is well borne by healthy digestive organs, but in certain cases of gastric disturbance it may give rise to temporary loss of appetite and dyspepsia, more rarely to nausea and vomiting, and possibly to diarrhœa.—*New England Medical Journal.*

**A New Method of Local Refrigeration by Chloride of Methyl.**—M. Bailly concludes, from numerous experiments, that plugs of which the centre is formed of dry cotton wool, and the periphery of floss silk—the whole surrounded by gauze—constitute the best agent for imbibing and preserving the refrigerant liquid. Two-thirds of cotton wool to one-third of floss silk are employed.

By a prolonged application of these plugs, saturated with chloride of methyl, M. Bailly has soothed the pain in 26 cases of toothache, and in 9 cases of facial neuralgia. Out of 10 cases of sciatica, he obtained successful results in 8 cases. In 62 cases of different forms of neuralgia recovery was almost invariably the result of this treatment. Of 16 cases of lumbago 14 were rapidly cured. M M. Dieulafoy, Bucquoy, Fereol, Lailler and Pozzi have employed this method to soothe visceral pain. M. Bouchard has found it efficacious in intercostal neuralgia, torticolis, muscular pains, lumbago, toothache, and in one case of lead colic and gastric attacks of tabetic ætiology. M. Bouchard found that at certain times it modified dyspnœa in an emphysematous, asthmatic patient.

M. Bailly has employed this method to obtain local anæsthesia before opening abcesses, incising panaris, removing cancroid, and in an operation for anal fistula.

M. Videl stated that he had performed over 120 operations of different kinds, after anæsthesia was obtained by M. Bailly's method, which was applied about 360 times in those cases in which the operation could only be completed in several *seances*.

M. Bouchard remarked that M. Bailly had perfected M. Debove's method. M. Bouchard has substituted the reaction produced by refrigeration for actual cautery with advantage, especially in the treatment of uterine affections. M. Besnier believed that M. Bailly's method might prove of great service when applied to the mucous membranes, but added that it would be advisable to interpose a piece of plaster.

A vote of thanks to M. Bailly for his perfection of a valuable method of local refrigeration was proposed by Dr. Vidal, and was unanimously adopted.—*N. O. Medical Journal.*

**Typhoid in Children.**—Dr. Forcheimer, (Cincinnati), said in a Clinical lecture: He believed in the possibility of aborting typhoid fever with calomel, and thought he had done it six times during this epidemic, and that he had done it before. The whole trouble lies in the inability to prove this. If I get a case before the fifth day, I always give a dose of calomel, and a large one, sometimes repeating it. I follow this up with a rather full dose of antipyrine because it lessens pain and seems to have an antiseptic effect. I consider it of great importance to have two beds, one for the day and one for the night. It is necessary to have, the largest, lightest and airiest room in the house. The diet should be absolutely fluid. No bread, no toast. I have seen hemorrhages occur from bread. Thepatient will complain bitterly of having nothing solid to chew. Inthese instances I find it very satisfactory to give tolu to chew. I frequently give a drop of the dilute nitro-muriatic acid every hour. I give sustaining measures, mainly whisky, and in antipyretics avoid everything which will cause a collopse. I give the luke-warm bath.—*N. O. Medical Journal.*

**Antipyrine in Labor.**—At the meeting of the French Academy of Medicine, March 13, 1888, a paper by Dr. Queirel was read, in which he claimed that antipyrine is a valuable drug in labor, as it calms the excitement of the parturient woman and lessens her pain. Its influence is most beneficial in the first stage of labor; and it does not interfere with its progress at all. Dr. Queirel founds his opinion upon an experience of twenty cases, in fifteen of which his results were very satisfactory. He administers the drug by (hypodermic?) injection, in doses of about four grains, giving a second injection in two hours if the first has not produced the desired effect. Usually he found the influence of the antipyrine to be manifest in from twenty to twenty-five minutes; and he compares it to that of chloroform, with the great advantage that it is a much safer agent.

The announcement of this action of antipyrine tends to confirm the growing opinion that it is an admirable nervine, and supports the views of Germain See and of Robin in regard to it.—*Med. and Surg. Reporter.*

**Lactic Acid in the Diarrhœas of Children.**—Dr. G. Hayem, more than a year ago, called attention to the remarkable utility of lactic acid in the diarrhœas of children. Recently, in a communication to the Academy of Medicine (*Review de Therap.*, February 15), he has renewed his suggestion, and presented new evidence of the value of the remedy. He finds that better results are had from larger doses than he formerly advised. In the more severe cases he has administered a 2 per cent. solution up to twenty teaspoonfuls in the course of twenty-four hours. The formula employed by him is the following:

| | |
|---|---|
| Lactic acid (pure) | ʒss. |
| Syrupi | ℥j |
| Water | ℥iij. |

The strength of this is about one minim to the teaspoonful. The quantity given will vary with the age of the subject and the nature of the attack. M. Sevestre, one of the physicians to the Children's Hospital, confirms the statements of Hayem regarding the therapeutic power of the remedy in question, and he also finds that a considerable quantity is required to effect the best results. The latest experience demonstrates that a teaspoonful of the 2 per cent. solution should be given every five minutes in the worst cases, and from this up to a teaspoonful an hour; the amount required varies with the conditions present.—*American Journal of the Medical Sciences*, April, 1888.

**A New and Reliable Remedy for Coccygodynia and Pruritus Ani.**—Dr. R. Stansbury Sutton, Pittsburg, Pa., in *Medical and Surgical Reporter*, says:

I have, for reasons I do not now care to speak of, regarded this disease as purely neurotic. I have treated it with the Faradic current. One treatment produces immediate relief; a few treatments cure it. Three cells are sufficient; time, five minutes; the frequency of application depends upon the return of pain. The anode is placed over the sacrum and the cathode in the vagina or rectum, or over the sphincter ani muscle. This treatment, so far as I know, is original with myself.

Much has been written of late concerning the treatment of pruritus ani. I desire to add my own suggestion. The best remedy I have ever found is the galvanic current; the quantity required need not exceed five milliamperes; the time of application five minutes. The relief is immediate, and the application once or twice daily is quickly curative. The anode is placed over the perineum or base of the scrotum and the cathode against the sphincter ani, or, if required, within its grasp, bringing all the pruritic surfaces between the poles. I claim to be the first, so far as I know, to suggest this remedy for the treatment of this disease. I will ere long have more to say of it.

